# Defining Normal Liver Stiffness Range in a Normal Healthy Chinese Population without Liver Disease

**DOI:** 10.1371/journal.pone.0085067

**Published:** 2013-12-26

**Authors:** James Fung, Cheuk-kwong Lee, Monica Chan, Wai-kay Seto, Danny Ka-ho Wong, Ching-lung Lai, Man-fung Yuen

**Affiliations:** 1 Department of Medicine, Queen Mary Hospital, The University of Hong Kong, Hong Kong, China; 2 State Key Laboratory for Liver Research, The University of Hong Kong, Hong Kong, China; 3 Hong Kong Liver Foundation, Hong Kong, China; 4 Hong Kong Red Cross Blood Transfusion Service, Hong Kong, China; 5 Access Medical Diagnostic and Laboratory Centre, Hong Kong, China; Yonsei University College of Medicine, Korea, Republic Of

## Abstract

**Background:**

For patients with chronic liver disease, different optimal liver stiffness cut-off values correspond to different stages of fibrosis, which are specific for the underlying liver disease and population.

**Aims:**

To establish the normal ranges of liver stiffness in the healthy Chinese population without underlying liver disease.

**Methods:**

This is a prospective cross sectional study of 2,528 healthy volunteers recruited from the general population and the Red Cross Transfusion Center in Hong Kong. All participants underwent a comprehensive questionnaire survey, measurement of weight, height, and blood pressure. Fasting liver function tests, glucose and cholesterol was performed. Abdominal ultrasound and transient elastography were performed on all participants.

**Results:**

Of the 2,528 subjects, 1,998 were excluded with either abnormal liver parenchyma on ultrasound, chronic medical condition, abnormal blood tests including liver enzymes, fasting glucose, fasting cholesterol, high body mass index, high blood pressure, or invalid liver stiffness scan. The reference range for the 530 subjects without known liver disease was 2.3 to 5.9 kPa (mean 4.1, SD 0.89). The median liver stiffness was higher in males compared with females (4.3 vs 4.0 kPa respectively, p<0.001). There was also a decline in median Lliver stiffness in the older age group, from 4.2 kPa in those <25 years to 3.4 kPa for those >55 years (p=0.001).

**Conclusions:**

The healthy reference range for liver stiffness in the Chinese population is 2.3 to 5.9 kPa. Female gender and older age group was associated with a lower median liver stiffness.

## Introduction

For people with chronic liver diseases, assessment of liver fibrosis is important for several reasons. Firstly, the degree of fibrosis is an indication of the severity of the underlying liver disease. Secondly, it may have prognostic significance. In addition, treatment decisions may be dependent on the presence of significant fibrosis. Although liver biopsy remains the gold standard for assessing liver fibrosis, patients may be unwillingly to undergo a biopsy procedure and clinicians may be reluctant to advocate it because of the potential adverse effects associated with this invasive procedure [1,2]. Furthermore, there are also disadvantages associated with liver biopsy. Its accuracy is dependent on the quality of tissue sample obtained, including the number of portal tracts, length of specimen, and degree of fragmentation. As biopsies only sample tiny portions of the liver, they are subjected to sampling errors. Its interpretation is further subjected to intra- and inter- observer variability [3]. For these reasons, non-invasive methods to assess liver fibrosis have been developed as an alternative to liver biopsy. 

In the past decade, liver stiffness measurement (LSM) using transient elastography has become one of the most viable alternative non-invasive methods to biopsy in assessing liver fibrosis. Previous studies have documented the diagnostic accuracy of LSM in grading fibrosis for a variety of chronic liver diseases [4-8]. In addition, LSM has been shown to have prognostic significance in predicting long-term outcome, and in the assessment of treatment response [9,10]. One of the earliest key concepts of LSM to evolve is that different optimal liver stiffness cut-off values correspond to different stages of fibrosis, and these cut-off values are disease-specific [4-7]. Although cut-off values for advanced fibrosis and cirrhosis have been well established for different diseases, the normal reference range of LSM in specific population groups have not been well defined, especially from large population studies. Histology from normal liver is rarely available, therefore normal ranges of liver stiffness is much harder to establish. An earlier small-scaled study using normal livers from subjects undergoing donor hepatectomy have identified a normal cut-off liver stiffness of <7.2 kPa [11]. 

The Hong Kong Liver Health Census was established in 2010 by a group of hepatologists from the University of Hong Kong in collaboration with the Hong Kong Liver Foundation and Hong Kong Red Cross Blood Transfusion Service. The aim of the current study was to define the normal ranges of liver stiffness in the healthy Chinese population without underlying liver disease.

## Subjects and Methods

The Liver Health Census recruited participants from blood donors of the Hong Kong Red Cross Blood Transfusion Services and volunteers from the general population. All participants included in the census were screened negative for hepatitis B surface antigen (HBsAg), antibody to hepatitis C virus (anti-HCV), and human immunodeficiency virus (HIV). Ultrasound of the abdomen and transient elastography was performed in all participants. Laboratory blood testing was performed on the same day. All participants underwent weight, height, and completed a detailed questionnaire. 

### Ethics statement

Written informed consent was obtained from all participants. The study was approved by the Institutional Review Board of the University of Hong Kong and Hospital Authority Western Cluster (No. UW-10-325).

### Liver stiffness measurement

LSM was performed using transient elastography (Fibroscan, Echosens, Paris) by an experienced operator. The procedure has been described previously [12]. Briefly, LSM was performed with the subject lying in the supine position. Using time-motion ultrasound image, measurements were obtained once a segment of the liver was located with a thickness of over 6cm and free of large vascular structures. Results were included in the final analysis only if the following three criteria were met: at least 10 valid measurements, success rate >60% and the interquartile range (IQR)-to-liver stiffness ration were ≤0.30. The median liver stiffness value of each participant was representative of the liver stiffness, and expressed in units of kilopascals (kPa).

### Abdominal ultrasound

All participants underwent abdominal ultrasonography by two experienced radiologists, using the Envisor ultrasound system (Philips Ultrasound, Philips Medical System, The Netherlands). Fatty liver was diagnosed by the presence of increased liver echogenicity. 

### Detailed questionnaire

All participants completed a detailed questionnaire including their past and current medical history, and their current intake of medications, herbal remedies, over-the-counter remedies, and alcohol.

### Laboratory test

Participants were excluded from the census if prior testing indicated they were infected with hepatitis B, hepatitis C, or HIV. Fasting blood tests were performed on the same day of the ultrasound and transient elastography. Liver biochemistry including alanine aminotransferase (ALT), gamma-glutamyl transferase (GGT) and bilirubin levels were measured, along with cholesterol and glucose. 

### Statistical analysis

All statistical analysis was performed using SPSS version 16.0 (Chicago, IL). Continuous variables with skewed distribution were analyzed using the Mann-Whitney test. Continuous variables with more than two categories were analyzed using the Kruskal Wallis test. The correlation co-efficient between LSM and other parameters were calculated using the Pearson test. The reference range was calculated using the mean ±2 standard deviations. A p-value of <0.05 was considered statistically significant. 

## Results

A total of 2,528 subjects were enrolled into the liver health census. As mentioned previously, these subjects were already screened negative for HBV, HCV, and HIV infection. To define a normal liver stiffness range in healthy individuals without known liver disease, we excluded also those with sonographical evidence of fatty liver disease (n=1070) and fibrosis/cirrhosis (n=4). Next, those with pre-existing diabetes, hypertension, other chronic medical conditions, and significant alcohol intake (as defined by >20g/day) were excluded (n=120). Those with elevated bilirubin (>17.1 umol/L), ALT (>45 U/L), GGT >84 U/L), fasting glucose (>6.0 mmol/L)[13], fasting cholesterol (>5.0 mmol/L)[14], and low albumin (<35 g/L) were then excluded (n=591). A further 207 patients were excluded with higher BMI (>23 kg/m^2^) or elevated blood pressure (systolic >140 mmHg or diastolic >90 mmHg)[15]. In the remaining 536, 6 (1%) subjects had invalid transient elastography measurements (as defined by the criteria set above), and were excluded. This is consistent with the predicted failure rate of <5%[16]. 

**Figure 1:**
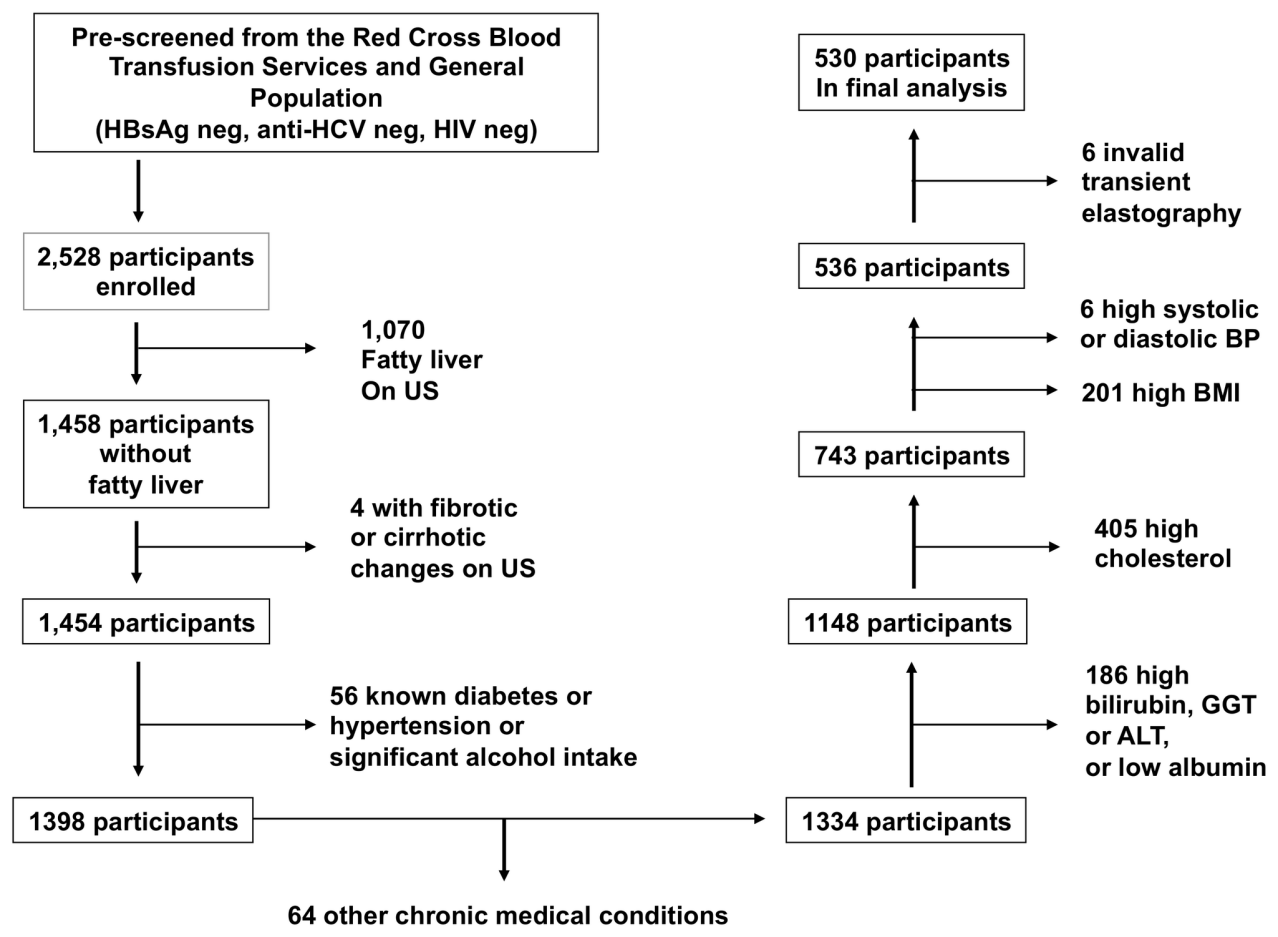
Flow diagram of health participants from the Hong Kong Liver Health Census.

The remaining 530 participants had valid LSM and were included in the final analysis. The flow of participants is outlined in Figure 1. Basic demographics and characteristics are summarized in Table 1. All patients in the final analysis therefore had normal liver parenchyma on ultrasonography, normal liver enzymes, absence of chronic medical condition, no significant alcohol intake, and normal fasting glucose and cholesterol and normal BMI. The laboratory and liver stiffness results are summarized in Table 2.

**Table 1:** Basic demographics and characteristics of healthy participants.

**Parameters**	**Values**
Number of participants (N)	530
Age (years)	37 (18-63)
Male sex [n(%)]	141 (26.6%)
Body mass index (kg/m^2^)	20.6 (16.0 - 23.0)
Systolic blood pressure (mmHg)	108 (80 - 139)
Diastolic blood pressure (mmHg)	66 (49 - 89)

Continuous variables expressed as median (range)

**Table 2:** Laboratory results and liver stiffness results of participants.

**Parameters**	**Values**
**Blood tests**	
Bilirubin (umol/L)	8.9 (2.1-16.9)
Alanine aminotransferase (U/L)	30 (16 - 45)
Gamma glutaryltransferase (U/L)	23 (9 - 73)
Albumin (g/L)	41 (36-50)
Fasting glucose (mmol/L)	4.8 (3.9 - 6.0)
Fasting cholesterol (mmol/L)	4.8 (2.6 - 5.0)
**Liver stiffness measurement**	
Liver stiffness (kPa)	4.1 (1.9 - 7.8)
Interquartile range	0.5 (0.0 - 1.6)
Success rate	100% (71 - 100)
Interquartile range to liver stiffness ratio	0.13 (0.00 - 0.30)

Continuous variables expressed as median (range).

### Correlation of LSM with patient parameters

**Figure 2:**
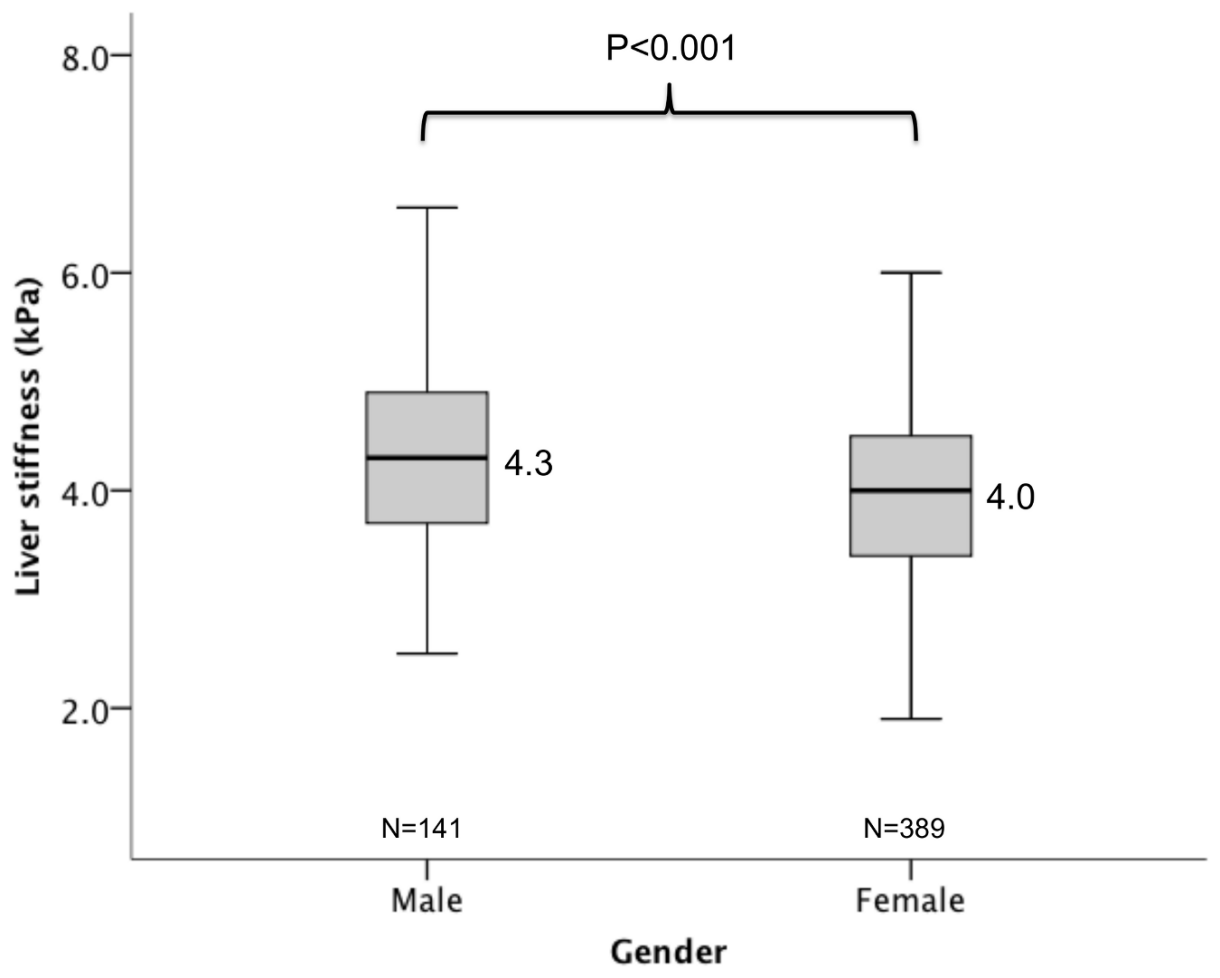
Liver stiffness according to gender.

**Figure 3:**
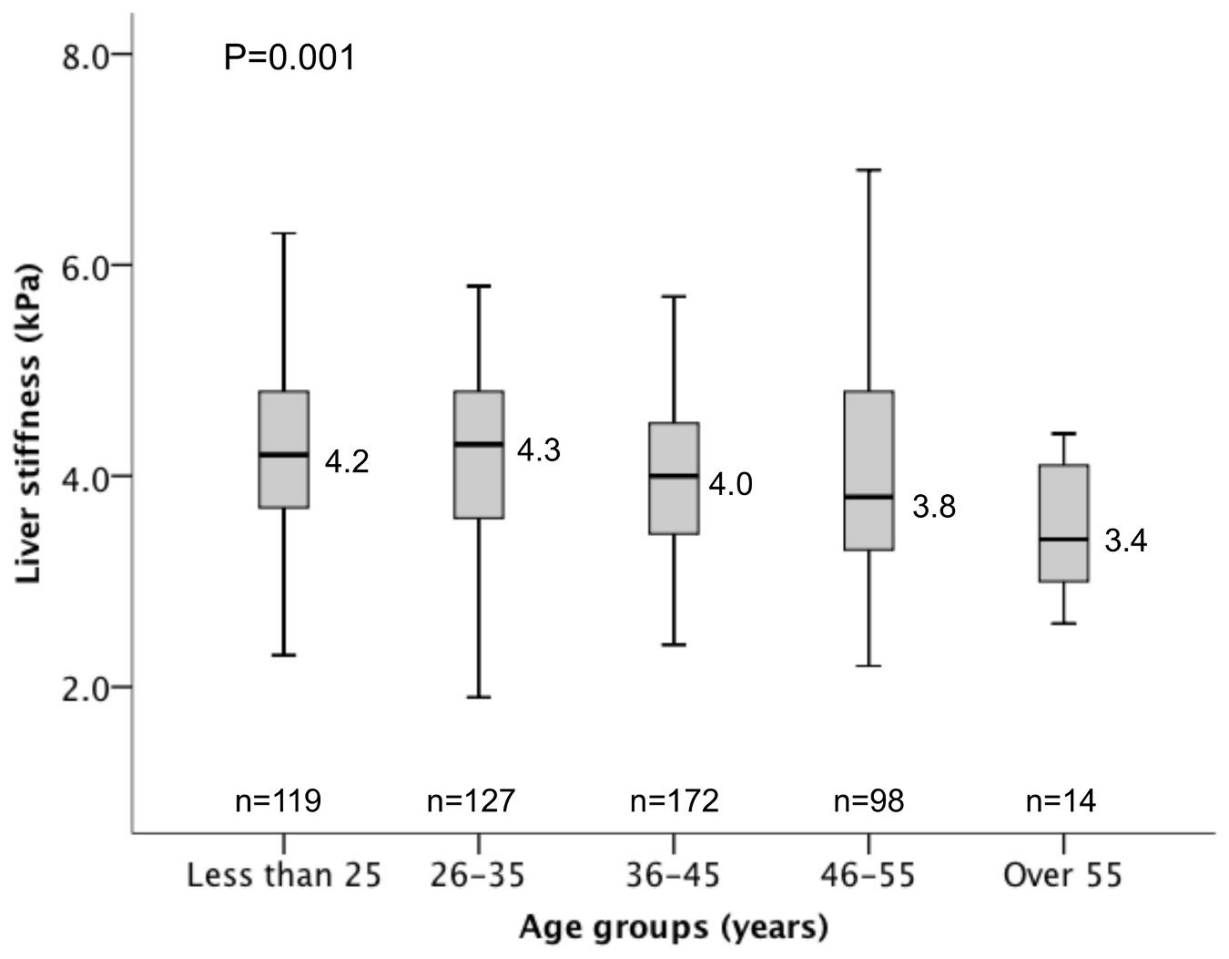
Liver stiffness according to different age groups.

There was a negative correlation between age and LSM (r=-0.168, p<0.001). The LSM for those with age ≤25, 26-35, 36-45, 46-55, and >55 years were 4.2, 4.3, 4.0, 3.8, and 3.4 kPa respectively (p=0.001), as shown in Figure 2. The median LSM in males was higher than females (4.3 vs 4.0 kPa respectively, p<0.001)(Figure 3). There was no correlation observed between LSM and BMI (r=-0.72, p=0.097), systolic blood pressure (r=0.061, p=0.162), and diastolic blood pressure (r=0.014, p=0.743). 

### Correlation of LSM with laboratory parameters

There was no significant correlation between LSM and fasting cholesterol (r=-0.61, p=0.163), fasting glucose (-0.78, p=0.071), ALT (r=0.072, p=0.100), and GGT (r=0.072, p=0.100). There was a weak correlation between LSM and bilirubin (r=0.096. p=0.028). 

### Normal reference range

**Figure 4:**
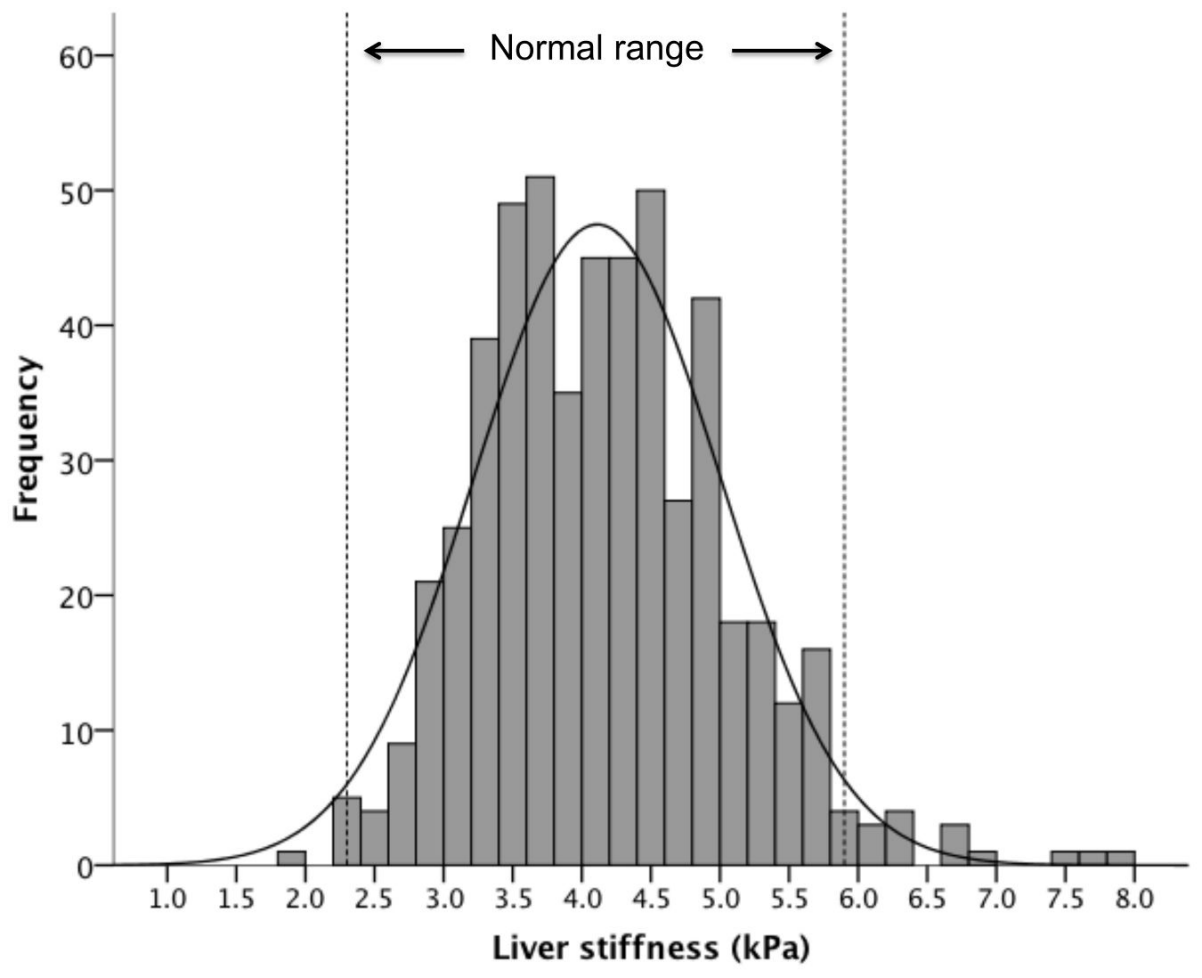
The distribution and normal range of liver stiffness in normal subjects.

The reference range for liver stiffness was determined for the current healthy population without known liver disease. The mean was determined, and the range of normal values was established by calculating ±2 standard deviations from the mean. The reference range for the 530 subjects without known liver disease was 2.3 to 5.9 kPa (mean 4.11, SD 0.89), with the distribution shown in Figure 4. The reference range was similar between males and females (2.6 to 6.1 kPa and 2.3 to 5.8 kPa respectively). 

## Discussion

In recent years, transient elastography has become more widely available for the non-invasive assessment of liver fibrosis. There are well-established optimal cut-off values for different stages of fibrosis, which appear to be specific for individual diseases and populations. Normal ranges for liver stiffness have been less well defined due to the lack of large population studies on healthy subjects. Histological specimens from normal livers in subjects without liver disease are expectedly rare. However, a large population study of normal healthy subjects is essential to derive a normal reference range of liver stiffness, which provides a solid and firm reference for populations with liver disease

The current study included participants from The Hong Kong Liver Health Census, selecting those without ultrasonographic evidence of fatty liver, fibrosis, and cirrhosis. In addition, none of these subjects were hepatitis B carriers, a disease that continues to be endemic with a prevalence rate of 8% in our general population. The study identified a healthy liver stiffness range of 2.3 to 5.9 kPa in the Chinese population. 

A recent study on 437 South Asian subjects showed a reference range to be 3.2 and 8.5 kPa by calculating the 5^th^ and 95^th^ percentile respectively [17]. A higher LSM was identified in lean and obese subjects. As the current study excluded subjects with high BMI (>23 kg/m^2^), no correlation between liver stiffness and BMI was observed among those with normal BMI, Another study of 445 South Asian subjects without known liver disease and normal liver enzymes showed a mean LSM of 5.1 kPa, with both ALT and BMI influencing the LSM significantly [18]. These differences highlight the important fact that liver stiffness values are not universal, and reference ranges should be derived for different populations. 

Although there was correlation shown between LSM and bilirubin, the correlation was very weak only. A previous large population study of 1,268 chronic hepatitis B Chinese patients demonstrated that LSM correlated with higher age and also increasing levels of ALT [12]. It has long been realized that ALT was a significant independent factor in which markedly elevated ALT levels can increase the LSM spuriously [19,20]. Another study also showed that even small increments in ALT were associated with higher LSM in CHB patients [21]. There was no correlation between liver stiffness and ALT in the current study. This is likely due to the fact that only those with normal ALT were included.

In a previous study of 428 patients with normal liver enzymes and no known liver disease, the liver stiffness was significantly higher in males compared with females [22]. This is also consistent with our current study showing a higher median stiffness in males compared with females (4.3 vs 4.0 kPa respectively, p<0.001). However, this difference is minimal, and therefore having separate normal ranges is unlikely to be useful.

One limitation of the current study is the unavailability of liver biopsies to confirm the absence of fibrosis in the study population. However, the use of liver biopsies in a large population of healthy individuals is unlikely to be feasible. By using stringent criteria to define a healthy population, the margin of error is likely to be minimized. Despite this, undiagnosed liver diseases may still occur. Although viral serology for HBV, HCV, and HIV were performed, neither hepatitis B core antibody (anti-HBc) nor autoimmune antibodies were screened to exclude past HBV infection and autoimmune liver diseases respectively. 

In conclusion, the normal range for liver stiffness in the Chinese population is 2.3 to 5.9 kPa. Female gender and older age were associated with lower liver stiffness in subjects without known liver disease.
